# Balance and fall-risk assessment in older adults using wearable plantar pressure and semi-supervised learning

**DOI:** 10.3389/fbioe.2025.1703500

**Published:** 2026-01-12

**Authors:** Jianlin Ou, Fangting Chen, Chengqiang Liao, Zhen Song, Lu Liu, Xiubao Song, Wei Bi, Liangliang Wang, Lin Shu, Zhuoming Chen

**Affiliations:** 1 Department of Rehabilitation Medicine, The First Affiliated Hospital of Jinan University, Guangzhou, China; 2 Department of Rehabilitation Medicine, The Sixth Affiliated Hospital of Sun Yat-sen University, Guangzhou, China; 3 School of Mathematics, South China University of Technology, Guangzhou, China; 4 School of Microelectronics, and School of EIE, South China University of Technology, Guangzhou, China; 5 Department of Biomedical Engineering, The Hong Kong Polytechnic University, Hong Kong, SAR, China; 6 School of Electronics and Information, South China University of Technology, Guangzhou, China; 7 Department of Neurology, The First Affiliated Hospital of Jinan University, Guangzhou, China

**Keywords:** balance assessment, elderly adults, fall risk, intelligent shoe, plantar pressure, semi-supervised learning

## Abstract

Falls are a major public health concern among older adults, leading to disability, reduced independence, and high healthcare costs. Conventional balance assessments such as the Berg Balance Scale are limited by subjectivity, time requirements, and dependence on trained evaluators, creating barriers for large-scale community application. To address these challenges, we developed an intelligent footwear system combined with a semi-supervised learning framework to objectively predict Berg Balance Scale scores and assess fall risk. In a study of 136 older adults aged 60–90, plantar pressure signals from smart insoles with eight sensors per foot were collected, and 156 biomechanical features were extracted. A multi-model error consistency approach was applied to mitigate label noise, and feature selection identified ten interpretable predictors related to pressure duration, peak intensity, and inter-limb symmetry. The model achieved root mean square errors of 3.99 in validation and 3.13 in an independent test group. This wearable-based, interpretable, and scalable approach provides a practical solution for early detection of fall risk, enabling timely community interventions and supporting healthy aging strategies in public health.

## Highlights

• Demonstrates a cross-disciplinary integration of wearable sensing, biomechanics, and machine learning for elderly balance assessment.
• Introduces a semi-supervised learning framework that mitigates label noise in clinical balance scales.
• Identifies biomechanical markers (pressure duration, intensity, symmetry) predictive of balance decline and fall risk.
• Provides an interpretable and scalable tool applicable to both clinical rehabilitation and community-based monitoring.
• Offers a translational pathway toward intelligent geriatric care and fall prevention strategies.

## 1 Introduction

The global population is aging rapidly, with the number of individuals aged 65 and older projected to double by 2050 (Luo et al., 2021). This demographic shift presents significant challenges to public health, particularly due to the increased prevalence of age-related health issues, including balance disorders and fall risk. Falls are one of the leading causes of injury and death among older adults, with an estimated 30%–40% of elderly individuals experiencing at least one fall per year (Xu et al., 2022). These falls often result in severe health consequences, including fractures, disability, and loss of independence, thereby placing a significant burden on healthcare systems worldwide (Tricco et al., 2017; James et al., 2020).

Clinical tools for assessing balance and fall risk, such as the Berg Balance Scale (BBS), are widely regarded as authoritative assessment methods (K, 1989; Shah et al., 2025). However, their practical application has certain limitations: reliance on subjective expert judgment, significant inter-rater variability, time-consuming procedures, and the fact that they are typically conducted in clinical settings, which makes it difficult to reflect real balance capacity during daily activities. Furthermore, data collected in clinical environments often contain inaccuracies in sample labels due to subjective and environmental factors. Directly using such data for model training can impair performance, while discarding it results in loss of valuable information. Therefore, there is an urgent need for more objective, efficient, reliable, and scalable methods to achieve real-time balance assessment in unconstrained daily environments.

The advent of wearable sensor technologies combined with advanced machine learning (ML) methods offers a promising solution to overcome these limitations, enabling objective, continuous, and unobtrusive monitoring of various physiological and biomechanical parameters related to balance and gait (Chen et al., 2022; Roshdibenam et al., 2021). Wearable sensors, including inertial measurement units (IMUs), smartwatches, fitness trackers, and smart clothing, can continuously collect data such as heart rate, acceleration, angular velocity, and plantar pressure. This data can then be processed and analyzed using ML algorithms, including support vector machines (SVM), K-nearest neighbors (KNN), artificial neural networks (ANN), and deep learning techniques, to provide real-time health assessments and predict fall risk (Moon et al., 2020; Subra et al., 2022).

Among the commonly used modalities, inertial measurement units (IMUs) placed on various body locations—such as the wrist, trunk, or lower back—have been widely adopted to capture motion data for gait analysis and fall risk classification (Hwang et al., 2024; Moghadam et al., 2023; Kiprijanovska et al., 2020; Nassajpour et al., 2024; Maiora et al., 2024; Sotirakis et al., 2024). For example, IMU-based systems have shown utility in detecting gait abnormalities (Kiprijanovska et al., 2020), estimating standardized balance scores (Nassajpour et al., 2024), and even predicting long-term fall risk in populations such as Parkinson’s disease patients (Sotirakis et al., 2024). Concurrently, plantar pressure systems embedded in insoles provide complementary insights into foot mechanics and weight distribution during gait, with applications ranging from identifying pathological gait patterns (Chen et al., 2021; Ekvall and Magnusson, 2013)to detecting loss of balance in occupational settings (Mfaa et al., 2018). Despite their respective strengths, both IMU and plantar pressure systems face implementation challenges, including sensor placement sensitivity, signal drift, and usability constraints in daily environments (Apas et al., 2024; Queen et al., 2010).

A critical issue common across these sensor-based approaches is their strong dependence on fully supervised learning paradigms, which require accurately labeled clinical data for training. In practice, the labels derived from widely used clinical balance scales such as the Berg Balance Scale (BBS) are often subject to noise due to inter-rater variability, subjective judgment, and contextual assessment limitations (Song et al., 2022). This label noise can significantly degrade model performance and generalizability, yet most existing studies continue to rely on conventional supervised frameworks without explicitly addressing this issue.

To address these challenges, this study explores an automated assessment approach based on wearable sensors and data-driven analysis. By incorporating a semi-supervised learning (SSL) framework, the system maintains robust performance even in the presence of label scarcity or noise, effectively leveraging suboptimal data quality and enhancing the model’s applicability in real clinical scenarios. Through the integration of algorithmic and sensing technologies, we aim to overcome the limitations of conventional methods, providing a clinically applicable and valuable solution for balance assessment while expanding the potential applications of wearable technology and data analytics in healthcare.

## 2 Methods

### 2.1 Study design

**FIGURE 1:**
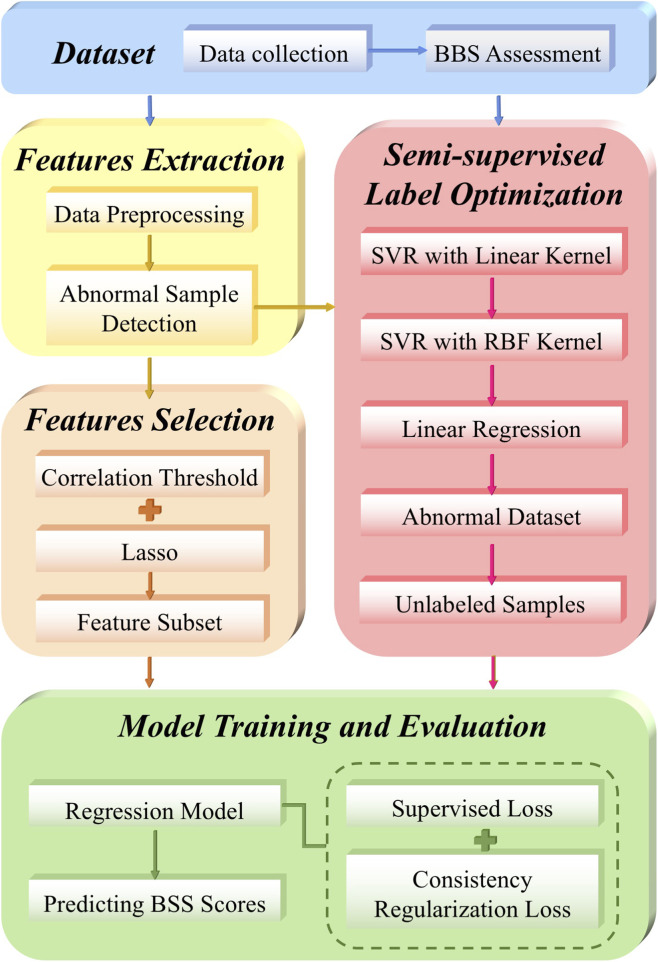
Algorithm flowchart. The workflow includes five key stages: data collection and BBS scoring, feature extraction, semi-supervised label optimization, feature selection, and model training and evaluation.

The aim of this study was to develop a plantar pressure data-based model using machine learning techniques to predict Berg scores, thereby assessing balance ability in elderly adults. The study utilized 131 data samples for model training and conducted testing on 29 individual samples independent of the training group. The research process comprises five main stages: dataset, feature extraction, semisupervised label optimization, feature selection, and model training and evaluation. The algorithm flowchart is illustrated in Figure 1.

### 2.2 Study subjects

Study volunteers were selected from hospitalized elderly patients, with the following inclusion criteria: 1. Age ≥60 years; 2. Normal vision, capable of independent walking lasting more than 3 min; 3. Clear consciousness, intact cognitive function, and ability to cooperate with assessments. Key exclusion criteria comprised major conditions known to significantly impair gait, including severe cardiopulmonary diseases, malignant tumors, diagnosed neurological disorders (e.g., stroke, Parkinson’s disease, peripheral neuropathy), severe musculoskeletal impairments, and recent lower limb surgery. Patient basic information is shown in Table 1, All participants signed informed consent forms and underwent comprehensive health assessments prior to the experiment.

**TABLE 1:** Participant demographics.

Classification		Training group	Test group
Number of participants		106	29
Gender	Male	64	21
	Female	42	8
age (years)		71.89 ± 7.37	70.04 ± 8.94
Body Mass index (kg/m^2^)		22.527 ± 3.17	22.85 ± 3.38
Comorbidities	Hypertension	59	15
	Diabetes	41	9
	Osteoporosis	12	6

### 2.3 Dataset

The experimental data consisted of three subsets. The first subset was obtained by sampling 84 older adults with varying balance abilities, yielding a total of 84 samples; this subset was intended to ensure model generalizability. The second subset was acquired through multiple sampling sessions involving 22 older adults, resulting in a total of 47 samples; this subset was intended to ensure model consistency. The third subset comprised 29 samples collected from 29 randomly selected older adults. In this study, the first and second subsets were combined to form a training set of 131 samples for model training, while the third subset of 29 samples served as an independent test set for model validation.

The plantar pressure was measured using a wearable insole system co-developed by the Human Data Science and Engineering Center of South China University of Technology and Zhongshan Yougan Technology Co., Ltd. (Song et al., 2022; Wu et al., 2022). Each insole was equipped with eight piezoresistive pressure sensors positioned at standard anatomical locations (hallux, metatarsal heads, midfoot, and heel). Data were sampled at 20 Hz and transmitted wirelessly via Bluetooth to a smartphone application for real-time visualization and storage. Participants walked on level ground at their natural pace while wearing the shoes. To ensure anatomical consistency across different foot sizes, the relative positioning of sensors within each shoe size was standardized to align with key foot landmarks (Wang et al., 2021). Complete technical specifications of the sensor system, including materials, measurement range, and performance characteristics, are provided in Supplementary Material S2.

**FIGURE 2:**
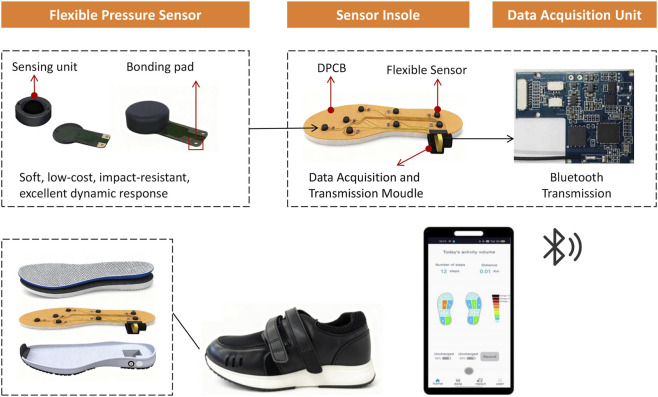
The workflow of the intelligent shoe system. System overview of the wearable intelligent shoe. The diagram illustrates the key components: sensor-embedded insole, data acquisition unit, central processor, wireless transmission module, and host-computer software for analysis.

**FIGURE 3:**
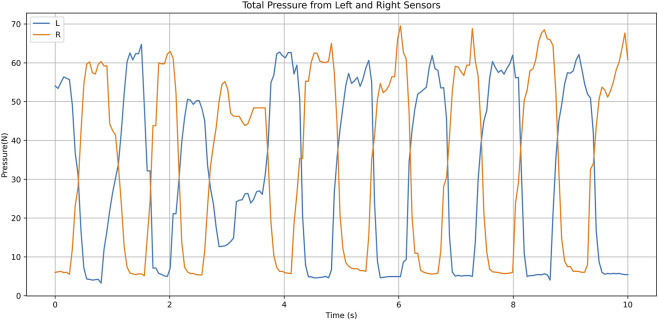
Pressure variation patterns of the L and R sensors over 10 secondsRepresentative time-series of total plantar pressure. The summed pressure from all eight sensors on the left (blue) and right (red) feet over a 10-s walking period is shown. The x-axis is time (s), and the y-axis is total pressure (N).

The shoe system transmits the collected data wirelessly via a Bluetooth-connected smartphone for real-time data updates. A dedicated mobile application dynamically renders plantar pressure distribution through vivid color gradients, mapped to distinct pressure levels, and enables real-time display, storage, and analysis of dynamic plantar pressure signals; the system was developed through Android Studio. Participants were asked to walk on level ground while wearing intelligent shoes for plantar pressure data collection. The experiment ensured participants walked naturally to capture authentic gait data. To ensure that plantar pressure data were comparable across participants with different foot sizes, we used multiple sizes of intelligent shoes. Each participant was fitted with the shoe size that matched their foot dimensions. Crucially, the relative positioning of the eight sensors within each shoe size is standardized to align with key anatomical landmarks (e.g., the heel, metatarsal heads). This design ensures that we are collecting pressure data from the same functional regions of the foot (e.g., the lateral heel, the first metatarsal head) regardless of the participant’s absolute foot length, thereby effectively controlling for this confounding factor. Figure 2 displays the shows the workflow of the intelligent shoe system, while Figure 3 illustrates the periodic pressure variation patterns of the L and R sensors over 10 s.

Following data collection, one licensed physical therapist with over 5 years of clinical experience conducted BBS evaluations for each participant. The BBS assessment comprises 14 items scored on a 0–4 scale, yielding a total score range of 0–56 points. During the evaluation, the therapists scored the participants’ performance according to standardized criteria and documented the composite scores.

### 2.4 Feature extraction

The feature extraction process consists of two main steps. First, a gait cycle detection algorithm based on Fourier transform and autocorrelation analysis is applied to identify the dominant period in the time series for each sample. Subsequently, the biomechanical feature values for each sample are computed according to the predefined feature parameter formulas. In this study, we extracted 156 key features from plantar pressure data to assess balance ability in elderly adults. The features included the plantar pressure peak value (PPP), pressure‒time integral (PTI), maximum and minimum pressure gradients, maximum pressure half-peak width (FWHM), area-based average pressure (AP), total average plantar pressure (TAP), forefoot/rearfoot pressure peak ratio (F/R), and symmetry index (SI) of the pressure distribution. Additionally, we calculated the total displacement of the X and Y coordinates for the center of pressure (COP) to further analyze postural sway changes. Detailed mathematical definitions for all features are provided in Supplementary Table S1.

Regarding the gait cycle parameter used in feature calculations, we developed an algorithm based on Fourier transform and autocorrelation analysis to detect the dominant period in the time series data. For the 16-dimensional plantar pressure time series, the raw data were first aggregated into a more representative two-dimensional time series by separately summing the pressure values from the eight sensors for each foot in real-time, resulting in total left and right foot pressure signals. Subsequently, period detection was performed independently on each dimension of this bivariate series. The detection algorithm integrated both Fourier transform and autocorrelation analysis to accurately identify the dominant gait cycle within the data. Finally, separate period values were obtained from the left and right total pressure signals. When these values matched exactly, the consistent value was adopted; when discrepancies occurred, the two periods were averaged and rounded to determine the final, unified gait cycle for each sample.

### 2.5 Feature normalization

**FIGURE 4:**
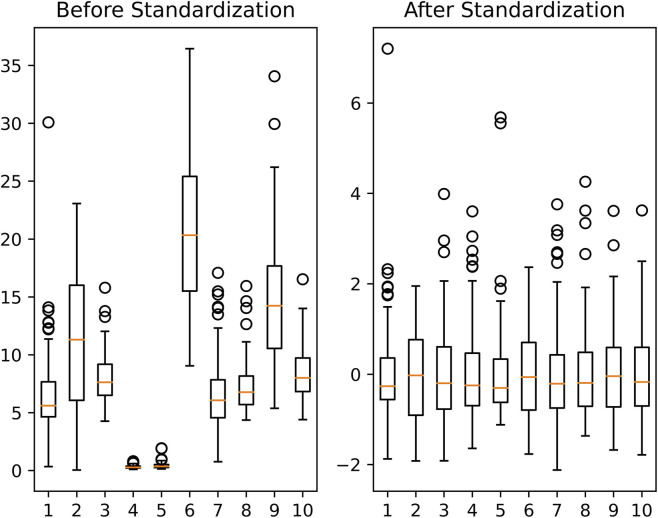
Comparison of parameter distributions in the dataset before and after standardization. The pre-standardization data exhibit substantial scale heterogeneity, whereas Z-score normalization successfully centers the distribution around zero with unit standard deviation, achieving scale invariance across all features.

To eliminate dimensional differences between feature parameters and improve model convergence efficiency, this study adopted the Z score normalization method based on a normal distribution for feature data standardization. Z-score normalization helps align the feature distributions closer to a standard normal distribution, thereby better meeting this underlying assumption and potentially improving model stability. Specifically, for the training group $X_{train}=\left\{x_{1},x_{2},\ldots ,x_{n}\right\}$ for each feature dimension *j*, its standardized value $z_{j}$ is calculated via Equation 1:

$$z_{j}=\frac{x_{j}-\mu_{j}}{\sigma_{j}}$$ (1)

where $\mu_{j}$ and $\sigma_{j}$ represent the sample mean and standard deviation of the *j*th feature dimension in the training group, respectively. The data distributions before and after standardization are shown in Figure 4.

### 2.6 Semisupervised label optimization

To identify samples with potentially noisy labels, we employed a trimodel ensemble comprising: Support Vector Regression (SVR) with a linear kernel, SVR with a Radial Basis Function (RBF) kernel, and standard Linear Regression. Each model was independently trained multiple times on the full training set (N = 131). For each sample, we computed a label-quality score as the mean absolute error between the model’s predicted and the ground-truth BBS scores across training repetitions, yielding three quality estimates per sample.

An error threshold was defined for each model as the 50th percentile of the absolute prediction errors. Samples whose error exceeded this threshold were flagged as candidate anomalies, resulting in N = 65 candidate samples per model.

The final anomalous sample set was determined by integrating the results from all three models. A sample was retained only if it was flagged by all three models. Through this consensus criterion, 42 samples were identified as possessing anomalous labels and were reassigned to the unlabeled set for subsequent semi-supervised training. These 42 samples were consequently removed from the labeled set and assigned to the unlabeled set. Importantly, their original BBS scores were discarded and not used as training targets in the subsequent learning phase.

### 2.7 Feature selection

To reduce multicollinearity among features while decreasing model complexity, we first calculated the Pearson correlation coefficient for each feature pair to construct a feature correlation matrix. One feature from each pair with absolute correlation coefficients exceeding 0.8 was considered a redundant feature and was screened out to enhance model stability and interpretability. Subsequently, feature selection was performed via L1-regularized LASSO regression. The optimal alpha parameter was determined through 5-fold cross-validation, followed by training the Lasso model on labeled samples via this optimal parameter. Through these two-step screenings, a final feature subset of size 10 was selected. The feature parameters in this subset exhibited minimum mutual correlations while representing the most predictive parameters.

### 2.8 Model training and evaluation

This study employs a consistency regularization linear regression model for training. With respect to loss function design, we constructed a composite objective function comprising a supervised learning loss term and a consistency regularization loss term

$$L_{\text{total}}=\underset{\text{Supervised Loss}}{\underbrace{\frac{1}{N_{l}}\sum_{i=1}^{N_{l}}{\left\|f_{\theta }\left(x_{i}^{l}\right)-y_{i}^{l}\right\|}^{2}}}+\;\;\lambda_{u}\underset{\text{Consistency Loss}}{\underbrace{\frac{1}{N_{u}}\sum_{j=1}^{N_{u}}{\left\|f_{\theta }\left(x_{j}^{u}\right)-f_{\theta }\left(x_{j}^{u}+\delta_{j}\right)\right\|}^{2}}}$$

where $x_{i}^{l}$ and $y_{i}^{l}$ are labeled samples and their labels, $N_{l}$ is the labeled sample quantity, $x_{j}^{u}$ is the unlabeled sample quantity, $N_{u}$ is the unlabeled sample quantity, $f_{\theta }\left(\cdot \right)$ is the neural network model, and $\delta_{j}$ is the perturbation term sampled from the zero-mean Gaussian distribution.

The supervised learning loss primarily ensures the model’s fitting capability for labeled data, whereas the consistency loss enforces prediction stability against data perturbations, thereby effectively exploiting the distribution information contained in unlabeled data. Model weights were updated through loss function minimization. The trained model was evaluated on an independent test sample set. The model was trained using a composite loss function. The supervised loss was computed on the 89 labeled samples. The consistency regularization loss was applied to the unlabeled set, which included the 42 samples with masked labels. This loss encourages the model to produce similar outputs for an unlabeled sample and its slightly perturbed version, thereby leveraging the feature data of these samples without relying on their potentially inaccurate labels.

## 3 Results

### 3.1 Results of abnormal sample detection

**FIGURE 5:**
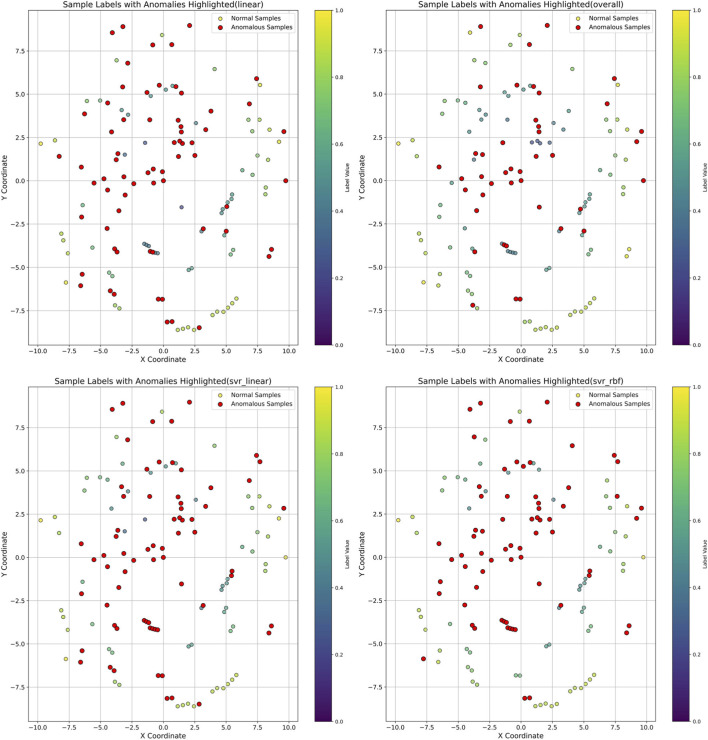
Abnormal sample distribution diagram. Samples were distributed radially according to their label values, with angular coordinates assigned randomly. The results show a high degree of overlap among the samples identified by different screening methods.

Figure 5 (upper left, upper right, and lower left) shows the identification results of abnormally labeled samples via linear regression, SVR with a linear kernel, and RBF-based SVR models, respectively. The radial coordinate magnitude and the color bar on the right together reflect the magnitude of the sample’s true label value. Samples with smaller true label values were more susceptible to identification as abnormally labeled samples; this aligns with the characteristics of the BBS assessment scale, where higher measurement errors exist in lower score ranges, and its scoring is more sensitive to subjects’ state fluctuations and assessors’ subjective biases.

**FIGURE 6:**
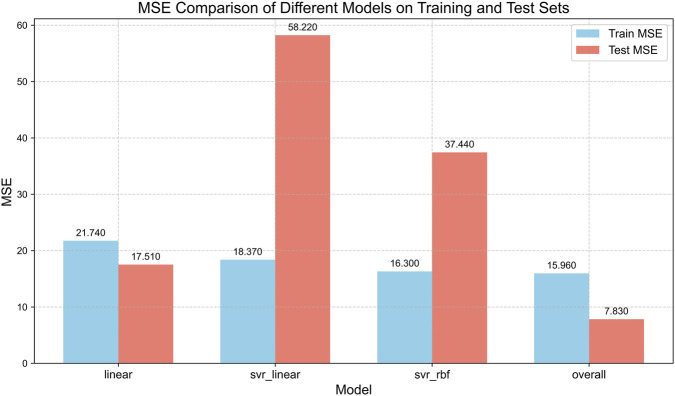
Mean Squared Error Comparison Diagram. Comparative performance of linear models on different filtered datasets.

The abnormal samples identified by the three models exhibited significant overlap. To ensure the detection accuracy and balanced numerical distribution of the sample labels, we comprehensively integrated the recognition results from all three models. (Manual Selection Method: Within the randomized framework, four replicate experiments were conducted for each of the three models, yielding a total of 12 abnormal sample sets. The frequency with which each sample appeared in these abnormal sets was recorded. A sample was manually marked as an anomaly if it appeared in eight or more of the abnormal sample sets. Through manual selection, a portion of the samples were ultimately chosen as the final abnormal sample set (N = 42), resulting in the detection of 42 labeled abnormal samples; this is illustrated in Figure 5 (lower right). Figure 6 shows the comparison of the mean squared errors for the four recognition outcomes on both the training group and test group. Figure 6 presents the performance of the models trained on the training group and test group using anomalous sample sets selected by three individual models versus those manually curated. The results demonstrated that, compared with experiments employing anomalous sample sets (N = 65) selected by the three individual models, the manually curated anomalous sample set (N = 42) enabled the final model to achieve superior generalizability.

### 3.2 Feature analysis

**FIGURE 7:**
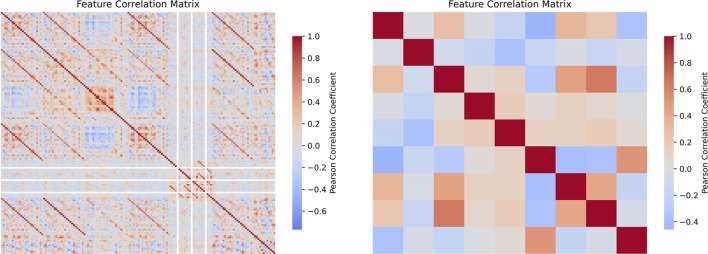
Heatmap of feature correlations before and after feature selection. The results indicate that the selected features eliminated the original multicollinearity and exhibit weak linear correlations among themselves.

**FIGURE 8:**
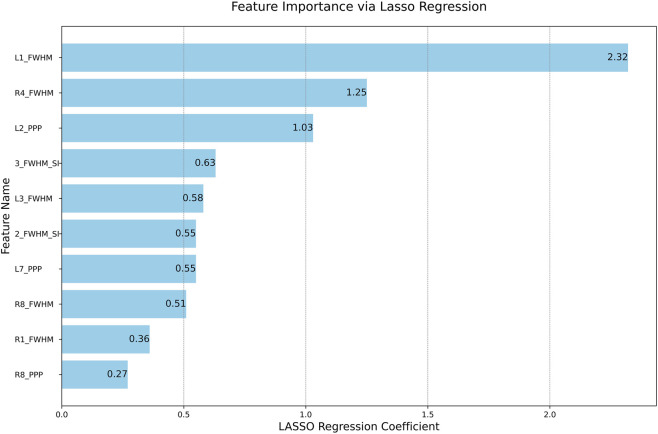
Feature weight distribution diagram. The bar chart shows the absolute values of the standardized coefficients for the 10 final selected features in the LASSO linear model. A larger coefficient indicates a greater contribution of that feature to predicting the BBS score.

Through rigorous screening and evaluation of feature parameters, we ultimately selected L1_FWHM, L2_PPP, R4_FWHM, 3_FWHM_SI, 2_FWHM_SI, L7_PPP, R1_FWHM, L3_FWHM, R8_PPP, and R8_FWHM as the feature parameters for model training. The correlation of the selected feature parameters is shown in Figure 7, where these parameters exhibited lower linear correlations ( $\left|r\right|<0.5$ ) than the raw feature set (r = mean ± Std), effectively avoiding the potential impact of multicollinearity on model performance. Further feature importance analysis is presented in Figure 8. The parameters L1_FWHM and R4_FWHM demonstrated significantly higher weight distributions of 2.32 and 1.25, respectively, indicating their stronger discriminative power in predicting target variables. This establishes a robust predictive foundation for the model.

### 3.3 Results of model performance evaluation

**FIGURE 9:**
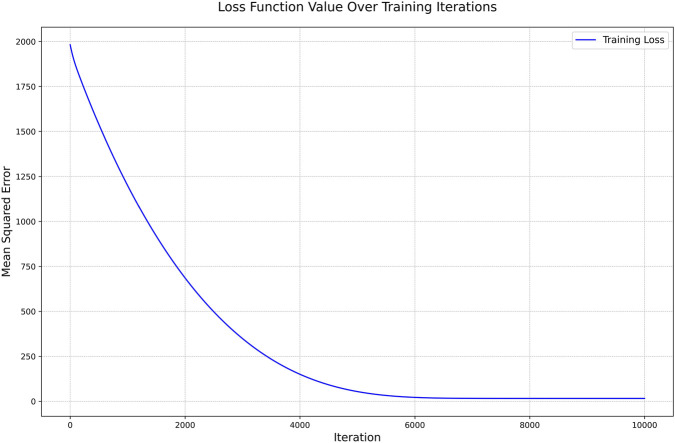
Variation curves of the loss function values. The total loss function (solid line) and the mean squared error (dashed line) decrease steadily with increasing iterations and eventually stabilize, indicating good convergence of the model training process.

The variation trend of the loss function designed with a composite objective function is shown in Figure 9, demonstrating convergence characteristics. The mean squared error exhibited a steady decreasing trend with increasing training iterations, eventually stabilizing. This monotonically decreasing convergence pattern indicates that the model parameters were effectively optimized and that the learning process was stable and reliable.

**FIGURE 10:**
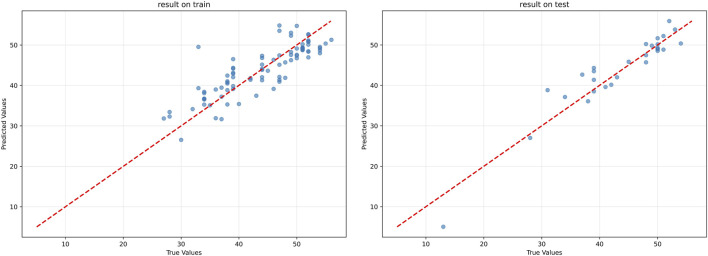
Comparison between true values and predictive values of the training group and test group. Left panel: results on the training group; right panel: results on the independent test group. Most data points are distributed near the reference line (y = x), indicating accurate predictions and good generalization performance of the model.

As illustrated in Figure 10, the errors between the predicted values and true values were generally maintained within a narrow range. For the training group, the root mean square error (RMSE) between the predicted values and true values was 3.99, whereas for the test group, the root mean square error was 3.13(For reference, a standard supervised regression model trained on the same data achieved an RMSE of 3.98 on this test set.). These results validate the effectiveness of the feature selection method and model architecture discussed earlier, providing a reliable foundation for the clinical application of the model. A comparison of the model evaluation parameters for the training and test groups is shown in Table 2.

**TABLE 2:** Comparison table of model evaluation parameters on training group and test group.

Data group	RMSE	MAE	R2
Training group	3.99	1.79	0.8398
Test group	3.13	1.54	0.9361

## 4 Discussion

### 4.1 Regression results and their implications

A linear regression model was employed within a semisupervised framework incorporating both supervised loss and consistency loss. The model achieved RMSEs of 3.99 and 3.13 on the training group and test group, respectively. These results demonstrated that the model effectively fit the training data and also had strong generalizability. The lower RMSE on the test group than on the training group further indicated that the introduced consistency loss plays a critical role in stabilizing model learning and enhancing adaptability to unseen samples. The superior performance of our SSL model arises from its principled handling of label uncertainty inherent in clinical scores. The framework strategically decouples two learning objectives: it uses high-confidence labels to establish accurate regression relationships, while repurposing samples with ambiguous labels as unlabeled data. The consistency regularization term then leverages the feature distribution of these unlabeled samples to enforce a smoother, more stable prediction function, effectively transforming potential label noise into a source of robust generalization.

Moreover, despite the simplicity of linear regression, its robust performance in this study suggests that, in machine learning tasks with limited training samples, preliminary data cleaning, sample quality control, and the effectiveness of feature engineering are more crucial than are complex model architectures. Consequently, in scenarios with constrained medical resources and limited availability of data and labels, future research should prioritize optimizing data and feature quality rather than solely pursuing model complexity and functionality, as excessive complexity may compromise interpretability and lead to overfitting.

### 4.2 Feature engineering results and their significance

Each sample in the original dataset comprised 156 feature parameters. Directly employing these for model construction could not only lead to high model complexity due to dimensionality but also introduce severe multicollinearity issues, thereby compromising model stability and interpretability. More critically, these features were derived from formulas with well-defined physical significance. Consequently, dimensionality reduction techniques such as principal component analysis (PCA), despite their advantages in data compression, may obscure the intrinsic physical meaning of the features, complicating model interpretation (Bomrah et al., 2024).

To address these challenges, a three-step feature selection strategy was implemented: Manual review: features deemed clearly unreasonable (e.g., min_PG-related parameters) were excluded; correlation analysis: highly redundant features were removed, retaining only the most representative variant; and LASSO regression: key features were further ranked and selected, ultimately yielding 10 parameters with strong representativeness and predictive power.

Although full-feature modeling was infeasible due to the high dimensionality of the original dataset, the model trained on this refined feature subset demonstrated excellent test performance, indirectly validating the efficacy of the selection approach. To ensure interpretability and clinical relevance, the 10 retained features were systematically categorized and analyzed. These features primarily fell into two distinct classes:

FWHM-related features: Including L1_FWHM, R4_FWHM, R1_FWHM, L3_FWHM, and R8_FWHM, the FWHM reflects the duration during which a specific region maintains high pressure, effectively characterizing the stable contact process between the foot and the ground. As a temporal parameter of plantar pressure distribution, the widening of the FWHM indicates prolonged stability of the COP during the stance phase. During heel strike, increased FWHM is associated with prolonged activation of the tibialis anterior muscle, facilitating shock absorption upon foot contact (Robb and Perry, 2024). Conversely, during forefoot push-off, a prolonged FWHM corresponds to increased cocontraction of the gastrocnemius–soleus complex, improving propulsion moment stability (Warabi et al., 2020).

A wider FWHM typically signifies greater stability during the stance phase, particularly in the heel strike and forefoot push-off phases, demonstrating robust balance control. Clinical studies have shown that elderly individuals with poor balance often exhibit asymmetric or excessively short stance durations, leading to gait instability and increased fall risk (Si et al., 2024; Wang Gzwp, 2025).

PPP-related features, including L2_PPP, L7_PPP, and R8_PPP, reflect the maximum pressure exerted on localized regions and are influenced by individual weight distributions and muscular control. For example, R8_PPP, located in the lateral heel region of the right foot, plays a role in providing stabilizing support during initial contact (Duan et al., 2022; Sanchis-Sales et al., 2024). In individuals with well-coordinated gaits, the PPP in this region typically falls within a moderate range, reflecting an effective landing mechanism. In contrast, an abnormally high PPP may indicate body imbalance, leading to excessive pressure on a specific foot region or abnormal loading due to compensatory mechanisms (Li et al., 2024).

Additionally, two features reflect plantar pressure symmetry: 3_FWHM_SI and 2_FWHM_SI. The SI is a critical metric for evaluating bilateral foot coordination (Ekvall and Magnusson, 2013). Research has demonstrated that increased fall risk in elderly individuals is often associated with significant functional asymmetry between the feet, particularly in cases of muscle weakness or diminished neuromuscular control (Nedovic et al., 2024).

Rationale for Redundant Feature Elimination and Advantages of Representative Feature Selection: During correlation analysis, certain features exhibited high collinearity (r > 0.8) in describing pressure distribution within the same region, such as between L1_FWHM and L1_PTI, as well as between L2_PPP and L2_AP. This phenomenon arises because these features are inherently derived from the same raw pressure curve, differing only in their temporal or spatial representations. To mitigate multicollinearity effects, we retained the metrics that demonstrated superior predictive performance and greater interpretability in terms of biomechanical significance. For example, L1_FWHM was selected as the representative feature over L1_PTI because it more clearly reflects the duration of stable contact during the gait cycle and has a greater weight in the training model.

Consequently, the final set of 10 selected features primarily encompasses three core categories: plantar pressure duration, peak intensity, and symmetry. These features ensure robust predictive performance (as evidenced by favorable model performance metrics) and also retain strong physical interpretability and clinical relevance, aligning with this study’s feature engineering objective of enhancing interpretability while reinforcing model stability. Furthermore, our choice of Z-score normalization over alternatives like min-max scaling enhanced robustness to potential outliers in clinical plantar pressure data and better satisfied the normality assumptions underlying our linear modeling approach. This feature selection strategy effectively reduces input dimensionality and also improves model training efficiency and interpretability, ensuring that the model’s predictions remain physiologically plausible.

### 4.3 Effectiveness and significance of semisupervised methods

During the initial phase of the study, the original dataset employed was fully supervised, with all the samples annotated with BBS scores. However, a critical experimental phenomenon was observed during model training: certain samples consistently exhibited larger prediction errors across multiple models. The residuals of these samples during training were generally greater than those of the other samples, with no discernible pattern in the error direction. The higher prevalence of prediction errors in the lower BBS score range points to the issue of label noise. In our semi-supervised framework, samples identified as ambiguous in this manner are processed by masking their labels and incorporating their biomechanical data into the consistency regularization loss, which helps to diminish the influence of potentially unreliable labels on the model. Directly incorporating these low-confidence label samples into the training process inevitably introduces label noise, leading to model learning bias and an increased risk of overfitting. To address this issue, this study developed a label quality discrimination mechanism based on multimodel error analysis combined with manual verification. Ultimately, 42 samples with anomalous labels were identified and reassigned to the unlabeled sample set, which was then incorporated into a SSL framework. Notably, this SSL approach achieved a test RMSE of 3.13, compared to an RMSE of 3.98 from a fully supervised model using the same initial data, indicating its potential for improved robustness against label noise. SSL leverages the strong supervisory guidance of labeled data and the distribution generalization capability of unlabeled data, making it particularly suitable for medical datasets characterized by limited sample sizes, high labeling costs, and nonnegligible label errors (Yang et al., 2025). In the field of rehabilitation engineering, SSL offers the following advantages:

Mitigating the Impact of Label Noise to Enhance Generalization Performance: In traditional supervised learning, erroneous labels directly lead to model parameter updates in incorrect directions. In contrast, under a semisupervised framework, mechanisms such as consistency regularization prevent the model from relying entirely on low-confidence labels. Instead, it guides the model to learn feature distributions under the supervision of high-quality samples, thereby improving robustness (Yang et al., 2024).

Enhancing the Modeling Capability for Boundary Samples and Clinical Sensitivity: In this study, samples with large prediction residuals were regarded as high-risk samples, which may represent transitional states near the falling threshold. In the semisupervised model, these boundary samples were permitted to participate in learning in a more flexible manner, thereby improving the model sensitivity to abnormal or transitional states; this aligns with the practical requirements of boundary risk identification in rehabilitation engineering.

Stable Performance Improvement Under Limited Sample Availability: Particularly in the medical field, where sample acquisition is costly and labels rely on expert assessment, semisupervised learning can effectively expand the training sample size by leveraging the underlying structural information of raw data, thereby achieving stable performance improvement in small-sample scenarios.

Final Data Composition and Model Performance: In the final dataset, 131 training samples were included, among which 42 were identified as unreliable label samples and converted to unlabeled samples, accounting for approximately one-third. The semisupervised approach incorporating consistency loss introduces constraints on unlabeled samples during training, effectively mitigating the interference of noisy labels and enhancing model robustness and generalizability. This was reflected in the experimental results—the model achieved an excellent test-group RMSE (3.13) even under conditions involving unlabeled samples.

Practical Implications: This approach demonstrates the significant value of semisupervised learning in real-world applications where data acquisition is challenging and data quality cannot be fully guaranteed; it maximizes the utilization of available labeled resources and improves the model’s adaptability to data anomalies through fault-tolerant training.

### 4.4 Comparative analysis with relevant studies

Current research on wearable fall risk assessment can be categorized into two main paradigms: first, binary classification based on sensor features (such as plantar pressure and acceleration) to distinguish between fallers and non-fallers (Shahzad et al., 2017); and second, continuous prediction of clinical balance scale scores (e.g., Berg Balance Scale scores) (Bacciu et al., 2017). While these studies have demonstrated technical feasibility, they commonly face two key limitations: first, model training often relies on standardized laboratory test environments, which restricts applicability in natural walking conditions; and second, most approaches do not adequately account for label noise introduced by inter-rater variability in clinical scoring (see Supplementary Table S2 for a summary of related studies). Directly using these labels for supervised learning may compromise model robustness.

In response, this study introduces targeted improvements in method integration and noise handling. We developed an intelligent shoe system suitable for natural walking scenarios, enabling real-time continuous prediction of Berg scores to enhance ecological validity. More importantly, to address label noise, we incorporated a semi-supervised learning framework. Unlike the common practice of discarding anomalous samples (Li et al., 2016; Lajoie and Gallagher, 2004; Kim and Xiong, 2022), this framework treats samples with low prediction consistency as “label-ambiguous” and incorporates them as unlabeled data into training. By leveraging their feature information through consistency regularization, we improve data utilization efficiency (effective training data increased by 34.7%) and model generalization without relying on synthetic data (Wu et al., 2024). The final model achieved an RMSE of 3.13 on an independent test set, outperforming some existing regression-based predictions (Bacciu et al., 2017). Furthermore, the key biomechanical features output by the model (e.g., peak pressure, symmetry index) are clinically interpretable, facilitating personalized rehabilitation guidance.

By integrating natural context assessment, noise-robust algorithms, and interpretable features, this study advances wearable balance assessment toward greater reliability and clinical translatability, offering a feasible approach for dynamic fall risk monitoring in community and home settings.

## 5 Limitations

This study has several limitations. First, the small sample size may have introduced errors. Second, although all BBS scores were assessed by therapists with ≥5 years of experience, interrater variability due to subjective factors may have caused score discrepancies. Since most training samples were evaluated by a single clinician, the model’s predictions aligned most closely with the clinician’s standards. Thus, discrepancies may arise when other clinicians use the model, necessitating future training with multirater assessments for improved generalizability. Third, the plantar pressure data were sampled at a frequency of 20 Hz. While this rate was sufficient to capture the fundamental rhythm and overall magnitude of pressure changes for the purpose of predicting BBS scores in our elderly cohort, it may limit the temporal precision for resolving very rapid gait events, such as the exact instant of heel-strike or toe-off. Future iterations of the system employing higher sampling rates could provide more granular insights into the dynamics of gait initiation and termination, which may be particularly relevant for studying populations with more erratic or higher-velocity gait patterns. Finally, our label noise detection strategy, which relies on model residuals, may be biased towards flagging samples with genuinely low BBS scores as 'abnormal’. a more sophisticated, score-invariant method for label quality assessment should be explored in the future to fully eliminate this potential bias. Furthermore, the frequency threshold for defining the abnormal sample set was determined empirically. To enhance robustness, future work should employ nested cross-validation to tune such hyperparameters within an inner loop, completely isolated from the final test group evaluation.

## 6 Conclusion

In conclusion, this study provides a novel and effective tool for assessing the balance capacity of elderly people and also pioneers new applications for smart shoes in fall prevention and health monitoring. These results demonstrate that plantar pressure-based balance assessment is a promising new approach with potential clinical value. Future research will further optimize model performance and explore practical applications in clinical and community settings for broader implementation.

## Data Availability

The original contributions presented in the study are included in the article/Supplementary Material, further inquiries can be directed to the corresponding authors.
