# Supplementary material for: Balance and fall-risk assessment in older adults using wearable plantar pressure and semi-supervised learning
Source: Front Bioeng Biotechnol. 2026 Jan 12;13:1703500. doi: 10.3389/fbioe.2025.1703500 (PMC12832853; doi:10.3389/fbioe.2025.1703500)
Supplement: Supplementary file 1 [file Table1.docx]

**Table 1 Calculation Formulas**

| Feature Parameters | Calculation Formulas and Definitions |
| --- | --- |
| Plantar Pressure Peak Value$L_{i}\_PPP(R_{i}\_PPP)$ | The maximum pressure value of the contact point during the entire sampling process, formulated as  $L_{i}\_PPP(R_{i}\_PPP)=_{n\in\left[ 1,N \right]}^{max}P_{i}\left( n \right)\vert_{L(R)}$ |
| Pressure‒Time Integral $L_{i}\_PTI(R_{i}\_PTI)$ | The time integral of pressure at the contact point during the entire sampling process, formulated as  $L_{i}\_PTI(R_{i}\_PTI)=\sum_{n=1}^{N-1} {(P}_{i}\left( n \right)\vert_{L(R)}+P_{i}(n+1)\vert_{L(R)})*\Delta n/2$ |
| Maximum Pressure Gradient $L_{i}\_maxPG(R_{i}\_maxPG)$ | The maximum change in pressure values between two consecutive sampling instances at the contact point  $L_{i}\_maxPG(R_{i}\_maxPG)=_{n\in\left[ 1,N \right]}^{max}{\nabla P}_{i}\left( n \right)\vert_{L(R)}=_{n\in\left[ 1,N \right]}^{max}{[P}_{i}\left( n \right)\vert_{L(R)}{-P}_{i}(n-1)\vert_{L(R)}]/\Delta n$ |
| Minimum Pressure Gradient $L_{i}\_minPG(R_{i}\_minPG)$ | The minimum change in pressure values between two consecutive sampling instances at the contact point  $L_{i}\_minPG(R_{i}\_minPG)=_{n\in\left[ 1,N \right]}^{min}{\nabla P}_{i}\left( n \right)\vert_{L(R)}=_{n\in\left[ 1,N \right]}^{min}{[P}_{i}\left( n \right)\vert_{L(R)}{-P}_{r}\left( n-1 \right)\vert_{L(R)}]/\Delta n$ |
| Maximum Pressure Half-Peak Width $L_{i}\_FWHM(R_{i}\_FWHM)$ | Maximum Pressure Half-Peak Width of the contact point within one cycle  ${L_{i}}_{\mathrm{FWHM}\left( {R_{i}}_{\mathrm{FWHM}} \right)}=n_{2}\vert_{L\left( R \right)}-n_{1}\vert_{L\left( R \right)},$  $其中P_{i}{(n}_{2}{)\vert}_{L\left( R \right)}=P_{i}{(n}_{1}{)\vert}_{L\left( R \right)} =0.5\times L_{i}\_PPP(R_{i}\_PPP)$ |
| Average Pressure Area$L_{i}\_AP(R_{i}\_AP)$ | Average pressure data collected at the contact point within one cycle  $L_{i}\_AP(R_{i}\_AP)=1/N\sum_{n=1}^{N} P_{i}\left( n \right)\vert_{L(R)}$ |
| Mean value of total plantar pressure $L\_TAP(R\_TAP)$ | Time-averaged sum of pressures from eight sensors on one foot within a cycle  $L\_TAP(R\_TAP)=1/N\sum_{n=1}^{N} \sum_{i=1}^{8} P_{i}\left( n \right)\vert_{L(R)}$ |
| Anterior-posterior pressure peak ratio  $L\_F/R(R\_F/R)$ | Ratio of forefoot peak pressure to hindfoot peak pressure within one cycle  $L\_F/R(R\_F/R){=(}_{n\in\left[ 1,N \right]i\in\left\{ 1,2,3,4 \right\}}^{max}P_{i}\left( n \right)\vert_{L\left( R \right)}){/(}_{n\in\left[ 1,N \right]i\in\left\{ 5,6,7,8 \right\}}^{max}P_{i}\left( n \right)\vert_{L\left( R \right)})$ |
| Total displacement of center of gravity position $COP\_R\_X(COP\_L\_Y)$ | Total displacement along one coordinate axis for a single foot during the entire sampling duration  $COP\_R\_X(COP\_L\_Y)=\frac{\sum_{n=1}^{N} \sum_{i=1}^{8} P_{i}\left( n \right)\vert_{L\left( R \right)}X_{i}(Y_{i})}{\sum_{n=1}^{N} \sum_{i=1}^{8} P_{i}\left( n \right)\vert_{L\left( R \right)}}$ |
| Asymmetry coefficient $SI\_i\_f$ | Asymmetry coefficient of the same feature parameter between homologous points on left and right feet  $SI\_i\_f=\frac{\vert f(i)\_L-f(i)\_R\vert}{0.5*(f(i)\_L＋f(i)\_R)}$ |

Note: n represents the sampling point index of the plantar pressure signals; i=1,2,…,8 denotes the eight plantar regions; $P_{i}\left( n \right)$ represents the plantar pressure value at the nth sampling point of the ith region, where L denotes the left foot, R denotes the right foot, and N indicates the number of sampling points in the gait cycle. $\Delta n$ is the time interval between two consecutive samplings by a sensor. $i\in\left\{ 1,2,3,4 \right\}$ corresponds to the forefoot region (including the first metatarsophalangeal joint area, second and third metatarsophalangeal joint areas, fourth and fifth metatarsophalangeal joint areas, and hallux area, monitored by the first four sensors), $i\in\left\{ 5,6,7,8 \right\}$ represents the rearfoot region (comprising the medial and lateral midfoot areas and medial/lateral heel areas, monitored by the last four sensors), and $X_{i} \mathrm{and}Y_{i}$ denote the relative coordinates of the pressure sensors.

**Table 2 Research on Fall Risk Using Different Sensors**

| **Reference** | **Participant** | **Sensor system** | **SensorLocation** | **Model** | **Specificity** | **Sensitivity** |
| --- | --- | --- | --- | --- | --- | --- |
| Yu et al(2021) (1) | 85 | Triaxial  accelerometer | Spine (lower  back) | Lasso  regression | 79% | 74% |
| Song et al.(2022) (2) | 48 | Pressure sensors | Insole | DT  GBDT  AdaBoost | 75% | 100% |
| Wu et al.(2022) (3) | 48 | Pressure sensors | Insole | MhNet | 70.4% | 76.72% |
| Lin et al.(2020) (4) | 51 | IMU  (accelerometer  + gyroscope) | Feet, spine  (trunk), spine  (sternum),arms | Binary logistic  regression | 78.40% | 71.40% |
| Dierick, F et al.(2022) (5) | 73 | IMU  (accelerometer  + gyroscope) | Spine (L4  vertebrae) | Multiple  logistic  regressions | 95.9% | 29.2% |
| Kim et al., (2023) (6) | 102 | Microsoft Kinect | Markerless (camera-based) | random forest(RF) | 86.1% | 83.3% |
| Colagiorgio et al. (2014) (7) | 79 | Microsoft Kinect | Markerless (camera-based) | SVM | 91.3% | 80.2% |
| Juneau et al. (2022) (8) | 80 | Smartphone (IMU) | Posterior Pelvis | Random Forest (RF) | 90.6% | 48.1% |

1. Yu L, Zhao Y, Wang H, Sun TL, Murphy TE, Tsui KL. Assessing elderly's functional balance and mobility via analyzing data from waist-mounted tri-axial wearable accelerometers in timed up and go tests. *BMC Med Inform Decis Mak*. (2021) 21: 108. doi: 10.1186/s12911-021-01463-4

2. Song Z, Ou J, Shu L, Hu G, Wu S, Xu X, et al. Fall risk assessment for the elderly based on weak foot features of wearable plantar pressure. *IEEE Trans Neural Syst Rehabil Eng*. (2022) 30: 1060-70. doi: 10.1109/TNSRE.2022.3167473

3. Wu S, Ou J, Shu L, Hu G, Song Z, Xu X, et al. MhNet: Multi-scale spatio-temporal hierarchical network for real-time wearable fall risk assessment of the elderly. *Comput Biol Med*. (2022) 144: 105355. doi: 10.1016/j.compbiomed.2022.105355

4. Ma L, Mi TM, Jia Q, Han C, Chhetri JK, Chan P. Gait variability is sensitive to detect Parkinson's disease patients at high fall risk. *Int J Neurosci*. (2022) 132: 888-93. doi: 10.1080/00207454. 2020.1849189

5. Dierick F, Stoffel PL, Schutz G, Buisseret F. High specificity of single inertial Sensor-Supplemented timed up and go test for assessing fall risk in elderly nursing home residents. *Sensors (Basel)*. (2022) 22. doi: 10.3390/s22062339

6. Kim T, Yu X, Xiong S. A multifactorial fall risk assessment system for older people utilizing a low-cost, markerless Microsoft Kinect. *Ergonomics*. (2024) 67: 50-68. doi: 10.1080/00140139. 2023.2202845

7. Colagiorgio P, Romano F, Sardi F, Moraschini M, Sozzi A, Bejor M, et al. Affordable, automatic quantitative fall risk assessment based on clinical balance scales and Kinect data. *Annu Int Conf IEEE Eng Med Biol Soc*. (2014) 2014: 3500-3. doi: 10.1109/EMBC.2014.6944377

8. Juneau P, Baddour N, Burger H, Bavec A, Lemaire ED. Amputee fall risk classification using machine learning and smartphone sensor data from 2-Minute and 6-Minute walk tests. *Sensors (Basel)*. (2022) 22. doi: 10.3390/s22051749

**Smart Shoes Introduction**

The plantar pressure was detected using a wearable system developed in collaboration with the Human Data Science and Engineering Center of South China University of Technology and Zhongshan Yougan Technology Co., Ltd. (22, 23). the shoe system is visually indistinguishable from ordinary footwear, with only a charging port positioned on the lateral side. Inside the sensing insole of the system, eight pressure sensors are distributed across key anatomical locations under each foot. These sensors are composite piezoresistive flexible pressure sensors, primarily composed of carbon black and silicone rubber, designed to meet the requirements for long-term daily plantar pressure monitoring.

The pressure measurement range is 0–760 kPa, with stable frequency characteristics within 0.5–2 Hz. Data were acquired at a sampling frequency of 20 Hz to ensure the capture of detailed gait and pressure variation information. The system exhibits a response time of 90 ms, a highly sensitive pressure range of 0–40 N, and a sensitivity of up to 0.0107-1 N^-1^. It is capable of withstanding approximately 1 million stepping cycles and demonstrates short response time, wide measurement range, high sensitivity, and excellent durability.
